# A Report on One Hundred and Twenty Knee Joint Operations

**Published:** 1908-06

**Authors:** Michael Hoke, C. R. Andrews

**Affiliations:** Atlanta, Ga.; Atlanta, Ga.


### A REPORT ON ONE HUNDRED AND TWENTY KNEE JOINT OPERATIONS.*

BY MICHAEL HOKE, M. D., AND C. R. ANDREWS, M. D., ATLANTA, GA.

You will be pleased to know that I have no intention of wearying you with a prolonged, detailed report of the knee joint operations mentioned in the title of this paper. It is not often that the general practitioner has the opportunity to see the inside of the joints when affected with the various conditions which have necessitated the operations. As the general practitioner is usually the first one to come in contact with these cases, it seems to me that if I select certain ones from the number, describe the conditions found in the joints and state the results obtained, it may be of value to you. Ninety-seven of these cases were the results of toxic arthritis. I wish to take a few of these

*Read before the Georgia Medical Association, Fitzgerald, April 15-16-17, 1908.

and show in connection with the clinical history, the various degrees of inflammation of the joints in this process, from the slightly inflammed to those in which the process had made further advancement, in which extensive changes had taken place in the joint tissues with consequential deformity.

Case I. Miss H.—This young lady had had indigestion for a number of years before she was seen by me. When I first saw her, both knees were swollen, the left slightly, the right considerably so. There was fluid in the right joint. A diffuse thickening of the joint capsule was palpable, and in the upper, inner quadrant a small mass could be felt. She had been "rheumatic" for some time. The fingers were inflammed and she also had pains in the other joints. The right knee was slightly bent. Palliative measures helped her somewhat, but the right knee remained enlarged. Her family took her to Hot Springs. She returned with the knee still swollen. She was then content to have the operation.

The joint was opened and a small amount of fluid evacuated. The synovial surfaces were injected, showing minute ecchymosis, and the small villi were enlarged so that the synovial surfaces showed minute elevations. The margins of the cartilage of the head of the femur were injected. Some of the blood vessels here were as large as a pin. The surface of the hyaline cartilage, instead of being white and glistening, presented a very fine tracing of capillaries. The mass in the upper, inner quadrant was a localized thickening of the joint capsule in that area. This tissue was removed and the joint washed out and closed. The subsequent history of this case was tedious, the main factor being the gastro-intestinal disturbance manifested by nausea, vomiting, now and then much gaseous distention, and obstinate sonstipation. The knee joint itself continued to be painful for a number of months, though the swelling entirely disappeared subsequent to the operation. It was necessary for this patient to wear a brace for a number of months in order to maintain the anatomical position of the leg.

This was one of the toxic joints in the earliest stages of inflammation. A microscopic section of the tissues excised showed an inflammation which was characterized mainly by infiltration around the blood vesesls. This was one of the first joints operated upon. At that time the writer felt that the cause of the inflammation was some poison circulated in the blood. This patient has been arthritis now and then since, but the end result was a normal joint with normal function. This case illustrates the earliest beginning of the toxic inflammation of the joint. At the time this case was studied and treated, the nature of the toxic process was not known. However, it was thought to be in the intestinal canal, and the line of general treatment was based on this conception of the nature of the disease.

Case 2. Age 21.—For about a year the right knee had been sore, following an injury. The joint had been treated with plaster-of-paris without avail. At the time she was seen, it was in the same condition that it had been for several months. The knee joint contained fluid and felt flabby, the swelling being chiefly above the patella. The general condition of the patient was of interest. She had had abdominal pains for some time, so severe at times as to simulate appendicitis. The least exertion brought on fatigue, and her complexion was muddy. The joint pains were confined to the knee. She was nervous and slept badly. On entrance to the hospital, her temperature ranged between 98 and 100. She was kept in bed for several days on a fermented milk diet. Soon the temperature became normal, the abdominal pain disappeared and her complexion cleared up. Her general condition was also much improved.

The knee joint was opened and a quantity of fluid evacuated in which there were numerous coagulae. The margins of the cartilages were congested. The capsule of the joint was thickened about 3-8 of an inch. No tissue was excised. The joint was washed out and closed. Recovery was uneventful. Fifteen days after the operation there was 90 degrees of voluntary flexion, and she was allowed to go home. When she left her general condition was excellent; was not nervous; had no abdominal pains; the knee did not hurt and she walked quite well. Off and on for several months, during which time she carefully dieted and exercised, there was now and then a return of the abdominal pain and pain in the knee joint, though recovery at the end of several months was complete.

This is also one of the early cases, exhibiting not only the evidence of early inflammation, but the presence of coagulae in the joint, which, had they not been evacuated, would have certainly been an irritant to the joint, keeping up the exudate within and the joint pains. The study of the stools and urine disclosed the usual evidence of chonic intestinal putrefaction. It illustrates also what we have often observed in these joint cases, in which the patient suffers profoundly from toxemia, that attacks simulating appendicitis are common; as is also appendicitis itself. There have been ten cases out of a hundred joint cases in which the appendix had been removed, and in which there was no doubt about the involvement of the appendix.

Case 3. Lady, 45 years old.—Several years previous to the time that I first saw her, she had had attacks of sciatica which would confine her to the bed for a short period. Then the left knee began to give her trouble, so that in the course of time she began to have attacks of excruciating pain in the joint. A surgeon at her own home made a diagnosis of "floating bodies," and removed half a dozen of so-called "joint mice," which are cartilagenous bodies produced by the formation of cartilage in small polypi which drop loose from atrophy of the pedicle. This gave some relief, but the joint was still subject to attacks now and then. Often she would wake in the night and in turning over the joint would become locked, and she would be stricken with severe pain in the joint. She was obliged to walk with crutches. In the inner border of the patella there was something palpable which slipped under the finger when the joint was flexed and extended.

The joint was opened and a piece of cartilage, still hanging to a pedicle, half an inch long, was found at the site where something had been felt. This was taken out. The cartilagenous surfaces of the femur and patella were normal, but where the cartilage joins the osseous part of the end of the femur, there were minute osteophytes, about half the size of a match head, which studded this line. They were not large enough themselves to produce any mechanical disturbance in the joint and were, therefore, let alone. The capsule of the joint was diffusely thickened, though not to any great extent. It was therefore not disturbed. The joint was closed, and she made an uneventful recovery. This patient has been in good health, and with general care and dieting, is now in excellent condition. The only thing that has been done to her since the operation was to fit her with a belt for sacro-iliac motion, the latter keeping up a certain amount of backache. This patient's excreta showed positive signs of intestinal putrefaction.

This joint illustrates a little further progress of the process, at this stage being associated, not only with evidence of thickening of the soft tissues, but with a hypertrophic process at the junction of the cartilage and the osseous portion of the bone.

Case 4. Lady, 45 years old.—This patient had been subject to chronic arthritis for 25 years, and had visited the Hot Springs every year. Both of her knees were involved. She came under my observation for the reason that in attempting to cross the right leg over the left, the right knee became locked and she was unable to unlock it. She had a gastro-intestinal history. There were Hebedens nodes on the fingers. The locked knee was very much thickened and enlarged, and had a boggy feeling, and two osteophytes could easily be felt. Likewise, too, one could squeeze under the finger in palpating, things that felt very much like varicose veins, the characteristic feeling of fringes.

The joint was opened on both sides and immediately that the capsule was slit, there popped out into the wound, a mass of worm like fringes. There was a most intense congestion of the soft tissues. The capsule itself was 1-2 thick, this thickness being made up of fibrous tissue. The joint was divided up into compartments by adhesions which ran across from the anterior to the posterior surfaces. The margin of the femur was studded with osteophytes, and the locking of the joints was due to the catching of a large osteophyte on the external condyle of the femur with a similarly situated osteophyte on the margin of the external tuberosity of the tibia. These were chiseled off and motion was again possible. The fringes were all excised. A plastic operation was done on the capsule of the joint after this fashion. The external fibrous capsule was separated, and the synovial membrane proper was separated from the subsynovial mass of fibrous tissue which was carefully dissected up from the line of incision to the margin of the patella on one side, and from the incision to the attachment of the capsule on the external point on the other. The two surfaces were then plastered together, reducing in this way the extent of its original thickness and preserving the synovial surface intact. The recovery was uneventful, the joint, however, requiring massage and necessitating the use of a brace for several months. She returned about a year and a half afterwards with no thickening, no pain and a voluntary motion of about 70 degrees.

In this case we have exhibited further what may happen to the longer standing inflammations than in the cases previously described. One sees here a marked limitation of joint motion and the actual locking of the joint, the villi reported in the first case, having in this instance grown in size, varying in length from 1-4 of an inch to 2 inches. One sees the greater length of time necessary to restore the joint to its usefulness; also the permanence of the result, even in the advanced cases when the joint is freed from all of its impedimenta and again allowed to participate in its normal functions, this being motion without deformity and capacity to bear weight without pain. An interesting thing here was that previous to the operation, the left knee, which was not operated upon, was regarded by the patient as being her "good knee," and when she returned for her last visit she stated that the knee upon which the operation was done and which had been the "dreadful knee," was now the good knee. She was, however, unwilling to have the left knee operated upon though it needed it.

Case 5.—This patient had been subject to "chronic rheumatism" for many years, many joints having been involved. His toes were twisted out of shape, flexed and dorsally dislocated on the heads of the metatarsal bones. One ankle was swollen and both knees very much enlarged, boggy to the touch, presenting osteophytes and permanently flexed about 15 degrees. There was also palpable a hard mass. The right elbow was involved, being very much swollen. The fingers were enlarged. There was pain in the spine and under the shoulder blades.

The right knee was opened. There was fluid and coagulae in the joint. There were numerous adhesions and a great mass of fringes which I exhibit to you here to show the formation and enlargement of the normal villi. The capsule was thickened and there were masses of fatty tissue which had formed in the upper part of the capsule just in front of the last two or three inches of the shaft of the femur. These masses were excised. There were, relatively speaking, craglike projections of bone and osteophytes standing out from the margin of the patella and the margin of the condyles. In the patella groove the surfaces were smooth as they usually are in the joint where there is present the gliding of two bone surfaces. The hypertrophic enlargement usually takes place at the margins of the joint, as spoken of in the recitation of the above cases. The subsynovial tissue, dense scar tissue in nature, was removed after the method stated above. The hemorrhage was so great that it was necessary to gather up all margins of the incisions with through and through sutures, gathering up the blood vessels collectively rather than individaully as the flow was mostly an ooze. The margins were then united. A small drain was put in the joint. There was a tremendous amount of serus ooze following the operation, but the joint recovered without any trouble.

This case illustrates the formation of fibro-fatty tissue between the capsule in front of the shaft of the bone as well as the formation, to an extreme degree, of osteophytes, fringes, polypi, adhesions, coagulae and the outpouring of fluid. I am not able to state the result in this case, as the patient passed out from under my observation, not being willing to undergo subsequent operations on the other joints.

Case 6.—A lady with a gastro-intestinal history for twenty-five years. Several years previous to the time that she came under observation the left knee gradually swelled and went through a very acute inflammation, being very large and red, painful and flexed for a month. She was confined to her bed for two or three months. She was treated by an orthopedic surgeon in the north, the line of treatment being plaster-of-paris casts, burning with cautery, etc. At this time very little was known about knee joints of this type, and the treatment was the treatment then in vogue. When seen the joint had been permanently flexed somewhat for about two years. There was no fluid in the joint; there was no swelling except on either side of the patella tendon.

The joint was opened and there was a dense mass of fibro-fatty tissue, very vascular, on either side of the patella tendon. This was at the site at which the joint had been most painful. The surface of the external condyle had been eroded, and there was a flat place, larger than a quarter of a dollar, of ivory like consistency. The scar tissue was dissected out, the joint capsule being smaller then to this extent. Recovery was uneventful, thought it was necessary to treat the joint for four or five months. This patient was so much improved that one year ago she traveled all through Europe, walking as tourists do through galleries and sight seeing. When first seen she was bedridden. There is about 70 degrees of voluntary motion present, but the joint cannot be quite straightened. She walks with a scarcely preceptible limp.

This case illustrates the erosion of the cartilage that occurs sometimes in the progress, as likewise the formation of dense scar tissue.

Case 7.—This patient gave a gastro-intestinal history existing for a number of years. Several years previous, the left leg had been amputated just above the knee on account of a tubercular (?) infection of that joint. During convalescence the left hip joint became ankylosed so that she had an ankylosed stump on that side. Several months after the operation the right knee became swollen, and when seen it was enormous, boggy, very much swollen below the knee and much atrophied above the knee. The right ankle was also very much swollen. She was emaciated, and the complexion had that sallow, brownish hue which goes with intense albuminous putrefaction. The stools were very, very foul. She presented all the general signs of toxemia.

This joint was opened on either side of the patella. A double handful of angry, inflammatory tissue was removed. Wherever there was a crevice into which this tissue could protrude, it had formed. There was nothing nice about this operation; it was simply a hacking away of chronic, inflammed tissue, masses of fringes and polypi. The feature here which is presented and which is not presented in the other cases is this, that the lower end of the femur was so soft that the slightest pressure upon the cartilagenous surfaces of the end of the femur would indent it. It had the feeling as if the end of the femur were cystic. This was not disturbed. The joint was closed. A great deal of suffering followed the operation; every stitch hole abscessed. The stitch infections were attributed to the low vitality of the patient.

This stitch hole infection illustrates a point of extreme importance in the handling of toxic joints, namely, that these toxic patients all have lowered vitality and all are subject to inflammation of other parts of the body, than joints, as is evidenced by the presistent presence of bronchitis, trachitis, pharyngitis, nasal infections, dactylitis, colitis, appendicitis and most every form of —itis that one might mention. The clinical meaning of it is this—that previous to the operation on cases which present evidences of toxic inflammation, one should be extremely careful to delay the operation, if possible, until the patient shall be placed in a suitable physical condition to stand it. I have operated upon cases in the sixties, who, when they presented themselves for treatment were in a state of extremely low vitality, and who, after they had been put on fermented milk and a soft diet and rested and slept in the fresh air for days or weeks as the case might be, until they were in good condition, their incisions would heal with as little disturbance as a scalp wound made under the best aseptic technique.

I exhibit here two knee joints which were the portions of a leg amputated by Dr. Armstrong several days ago, from a patient who had been a sufferer from arthritis deformans for a great many years, the operation being necessitated by gangrene of the feet. These specimens show the most extreme destruction which takes place around the joint in long standing process. You will see that the joints are entirely destroyed, distorted and deformed, that there is hardly any dense bone at all, the shaft of the bone and the end being simply a fine shell containing a mass of fat, and the spongy bone structure as coarse and about as dense as mosquito netting. Quite likely the condition of affairs at the lower end of the femur in the case last mentioned was about as seen here, plus the persence of enormous masses of highly inflammed tissue between the femur and the tibia as described above.

Case 8.—The following case will illustrate the formation of tumors in the knee joint. This class of cases is not so frequently seen as those with minute polypi and fringes, but they do occur. This woman, about 38 years of age, had had attacks of "rheumatism" in the past which were usually severe. Several months previous to the time she was observed, she had an attack of "rheumatism" in the left knee from which the knee did not recover, and the time when she first came under observation, the joint was enormously swollen, full of fluid and very tense, so much so, that nothing in detail could be made out by palpation. The swelling was chiefly above the patella. When the swelling is mainly confined to this locality it usually indicates the formation of a great deal of thickened tissue between the posterior capsular surfaces and the femur.

This joint was opened and about a pint of straw colored fluid removed. Loose in the joint was a tumor about one-half inch long and one inch thick, oval in shape. This was evacuated. There were membranous adhesions between the surface of the joint and the capsular space had been very much distended upwards so that the capacity of the joint was very much increased. At this site, at the upper border of the joint, there were masses of fibro-fatty tissue. This was not an isolated mass but had involved the capsule of the joint to such an extent that the upper border of the capsule was excised along with the mass. There were several small polypi which were also removed. The capacity of the joint was reduced by this excision of the excess in the upper portion, everything was sutured and the joint closed. Her recovery was uneventful, with a normal degree of motion and function without pain.

Case 9.—This case was that on an extraordinarily stout woman who had been suffering with so-called "rheumatic gout" for a great many years, presenting spurs on the tarsus and both knees being so extremely painful that she could not walk about the house, that she would wake in the night on account of this pain. The pressure of the bed clothes even caused sufficient pain to keep her awake. Her distress was extreme.

From both knee joints fatty tumors were removed which were attached to the joint surface by pedicles. These masses were not very large, one being about two inches long by one-half inch wide, irregular in shape. A microscopic section showed globules of fat with intervening fibrous tissue trabeculae in which numerous blood vessels ran, some with periarteritis and almost obliterated, and others showing a more recent periarteritis.

The interesting feature about this case was that she was rheumatic, using this word in the general sense, and that after the operation she would have attacks of acute pain in the points now and then, which would always be relieved by purgation and anti-rheumatic remedies, these measures previous to the operation not having any effect upon her at all. The case illustrates the formation of fatty tumors in the joint, and the relief afforded by operation and the hopelessness of general treatment when there are masses of tissue within the joint which can only be relieved through an excision. This group of cases illustrates quite well the condition of affairs most usually found in the toxic inflammations of the knee joint, and though there are a very much greater number of cases of this nature in the total number operated upon, yet these cases will serve to furnish the details of things found within the joint and the results to be obtained by operation. Of course, the operative procedures did not entirely terminate the treatment, in all instances there being a certain amount of post operative treatment varying in length of time from two weeks to one year. All that the operation can accomplish is to get rid of the products which, by their presence, are causing pain and interfering with the normal use of the joint. Subsequently to the operation it is necessary to utilize such measures as massage, manipulation, passive motion and apparatus to maintain the anatomical position of the joint while the leg is regaining its power of motion; to diet the patient properly and to maintain proper hygienic surroundings.

Case 10.—This case, in which there were several in the group, illustrates the formation of a good knee from a knee almost completely ankylosed from adhesions. This lady, a very large, stout person, stuck a pair of shears into the knee from which the joint was infected, this having lasted a number of months. At the end of this time the knee was bent almost to a right angle with extremely little motion present. She had considerable pain, particularly internal to the patella tendon when pressure was made at this site.

There was a mass of scar tissue just internal to the patella tendon which was removed by operation. While removing this, observation was made upon the character of the interior of the joint. The joint surface was entirely obliterated except the contiguous surface of the femoral condyle and the tibial tuberosity. The joint surfaces were adherent by a rather delicate grade of fibrous tissue. By manipulation, the patella was made movable, and by forcible flexion and extension and tenotomy of the biceps tendon, the amount of motion was increased. The joint was then sewed up. The subsequent treatment in this case consisted in the use of apparatus and massage, of passive hyperemia induced by superheated air. This extended over fourteen months and at the end of this time she was able to walk without apparatus and had voluntary flexion of 45 degrees.

One other case in this group illustrates a feature which is rather uncommon. This joint had been obliterated by gonorrheal infection. There was about 10 degrees of motion which was perfectly free, but at the end of the arc of this motion the joint would suddenly lock as if two obstructions came in contact with one another. The skiagram did not show any bone interlocking, so it was finally concluded that the limitation of motion was due to the presence of an extremely short patella tendon. This tendon was split lengthwise and the two ends spliced after the joint had been forcibly flexed. At the end of three weeks passive motion was begun with the production of voluntary flexion and extension, sufficient to do away with the stiff legged walk.

Out of seven cases of this nature, five were successful with the production of good motion of the joint without pain being experienced by this motion. The other two were failures.

I have operated upon one joint which presented bony ankylosis from so-called "rheumatism." The skiagram showed the union between the femoral condyle and the tibial tuberosity. The joint was opened on both sides, a flap dissected from the under surface of the tissue, and the joint chiseled loose. The joint was then sewed up in a slightly flexed position. Following the healing of the incisions there was slight motion, but considerable spasm when an effort was made to move the joint. An anaesthetic was administered and flexion producing 20 degrees of motion in the joint while under the anaesthetic, was obtained. This patient did not remain under observation, leaving the hospital one night with her belongings without saying "good-bye." I was never able to hear from her afterwards. It would have been extremely interesting to follow up this case as I believe that good motion could have been accomplished had she submitted to further after treatment.

Case 13.—This case illustrates a condition found in gonorrheal infection. This young man had both knees affected, having been observed after the disease had existed for six weeks. The right joint was swollen and contained a small amount of fluid as was evident by fluctuation, and the tissue on either side of the patella tendon felt boggy. The left knee was slightly swollen. It is of interest here to note that following urethral irrigation, the pain in the left knee would disappear, while it had no influence upon the right knee. It was decided that it was necessary to operate upon this joint.

The fluid evacuated was of a gelatinous nature, some of which looked as if it were softened coagulae, being mushy in consistence and quite clear. The surfaces of the joint were, of course, inflammed, being very much injected with small enlarged villi, not sufficiently enlarged to be removed. The normal ligamentam mucosum was very much enlarged, both in length and breadth, and the pouches on either side of the patella tendon were masses of recently formed granulation tissue. Part of the ligamentam mucosum was excised. The recently formed inflammatory tissue was excised also, and the joint cleared of all fluid and closed without drainage. Recovery from the operation was uneventful. It took about three months to regain the normal motion in this joint, and when last seen there was only a slight limitation of the normal motion, and the joint was normal in size, there being no thickening present.

Case 14.—One excision may be of interest. This was an old tubercular knee which had been incised in Italy by a very distinguished surgeon in Rome. The bones had been wired together. In the incision which I did, I first took off a small amount from the end of the femur, hoping to get away from the diseased tissue. The saw passed through good tissue for the most part, yet it bisected one area about one-half inch in diameter, with a cheesey centre. Wishing to get to a healthy surface, if possible, it was sawed a little farther back, and though good areas of bone were passed, the saw passed through a small focus as before. Another level was tried and another with the same result.

This illustrates that in chronic bone infections which have been going on for years, there are foci scattered throughout the bone with intervening areas of good bone. The patient had been in a very much depleted condition and did not do well. The incisions healed, but his general condition was still bad. On account of this the leg was amputated about a month afterwards. The recovery of his health was very rapid, so that in two months he was ruddy and strong again. I think it illustrates that in the chronic bone infections of this nature, where the body at large seems to be suffering from the bad effects of the local disease, it is better to get rid of the disease entirely than to try to preserve the joint which will certainly be a source of annoyance, and the probable source of a more wide spread disease.

There was one amputation on an old lady who had an inflammed knee, which was thought, at the time the joint was opened, to be a chronic toxic joint. Cultures showed staphylococcus aureus. The drainage which was established did not seem to relieve her of the general ill effect upon the body at large and amputation was advised on this account, though there did not seem to be, so far as the joint itself was concerned, any hurry for the amputation. That the amputation was wise was shown by the fact that the infection had extended up the thigh about five inches into the planes of the fascia below the muscles. Here again recovery followed rapidly upon the removal of the infected joint.

There still prevails in many quarters, a feeling that operations upon the joints are to be dreaded through fear of infection, through fear of possible ankylosis of the joints. I desire to say that the cavities of the joints can be invaded with even greater freedom than any of the other closed cavities of the body, that where there is no pre-existing infection of serious nature, you should not have the slightest dread of damage to the joint, and that increased motion is the rule rather than limited motion. I must acknowledge, however, that there is room for the display of the most varied grades of judgment, not only in the operative procedures that are done upon these joints, but in the subsequent management. I may say that in joint operations, without the most skillful judgment in the management of the post operative treatment of the joint, there is liability of failure in accomplishing the restoration of the joint function. For example —often times massage is extremely important during one week of the post operative treatment, when the same massage given at another week would do the joint damage and retard its progress. I may say that one must have familiarity with the personal characteristics of the joint. There is a something which is hard to describe which we can only know through intimacy with them, which enables one to be extremely conservative at one period of its management, and extremely radical at another period of its management, and only by the most careful attention to details in the management of them are they restored to usefulness.

---

The South Carolina Medical Association held its annual convention at Anderson, April 14 to 17 inclusive, under the presidency of Dr. Le Grande Guerry, Columbia.

